# Jaguar and puma captivity and trade among the Maya: Stable isotope data from Copan, Honduras

**DOI:** 10.1371/journal.pone.0202958

**Published:** 2018-09-12

**Authors:** Nawa Sugiyama, William L. Fash, Christine A. M. France

**Affiliations:** 1 Department of Sociology and Anthropology, George Mason University, Fairfax, Virginia, United States of America; 2 Department of Anthropology, Harvard University, Cambridge, Massachusetts, United States of America; 3 Museum Conservation Institute, Smithsonian Institution, Suitland, Maryland, United States of America; New York State Museum, UNITED STATES

## Abstract

From Moctezuma’s zoo to animals kept in captivity at Teotihuacan, there is increasing evidence that Mesoamericans managed wild animals for a myriad of purposes. The present study situates ritualized animal management of highly symbolic fauna in the broader context of Classic Mesoamerica by examining another core site, the Maya center of Copan, Honduras (A.D. 426–822). In this study, we identify two animal populations among the faunal remains from public and private rituals spanning the Copan dynasty. One population, with diets heavily composed of atypically sourced C_4_ inputs indicative of artificial feeding, corresponds with the felids interred in Altar Q and Motmot caches. The second population is composed of felids and felid products bearing a predominance of C_3_ signatures indicative of a more natural dietary regime. As with Copan deer, species-specific δ^18^O variations within these felid populations further substantiates the postulation that an expansive faunal trade network operated throughout the greater Copan Valley and beyond. Animals routed from sites of capture into the mesh of this network would have been processed into pelts, venison and other secondary goods or delivered alive to centers of state power for ritual usage and display. Our data reveal that at Copan, wild animals were routinely brought into intimate contact with human settlements to be managed and physically manipulated in a variety of ways in order to fulfill ritual and symbolic purposes.

## Introduction

Long before historical records mention Moctezuma’s zoo during the Aztec occupation (A.D. 1325–1521) [1], the earliest evidence of wild carnivore captivity in the New World was recovered at the site of Teotihuacan, Mexico (A.D. 1–550) during the rise of one of the largest urban metropoles in ancient Mesoamerica [2]. The apex predator sacrifices within the pyramid represented the culmination of a state ritualization process that entailed the effective physical and symbolic manipulation of key symbols (*sensu* Ortner) [3] of the Teotihuacan state [4]. The remains of these empowered carnivores, including golden eagles, pumas, jaguars, wolves, and rattlesnakes, help establish a rich zooarchaeological and isotopic baseline with which to investigate the extent of human-animal dynamics in comparable sites. The present study situates ritualized animal management of highly symbolic fauna in the broader context of Classic Mesoamerica by examining another core Classic Mesoamerican site, the Maya center of Copan, Honduras (A.D. 426–822).

Research on animal management strategies in the New World has been underemphasized largely due to the paucity of true domesticates in ancient Mesoamerica; a group represented in this region only by the dog [5] and the turkey [6–8]. In comparison to the substantial impact of Mesoamerican agricultural plants on the global economy during the Columbian Exchange [9], Mesoamerican animals were relegated to the status of mere zoological curiosities; a characterization which obscured the extent to which they were an essential component of daily life. Conversely, the intrusion of European livestock onto the Mesoamerican landscape had devastating effects on the ecosystem and transformed subsistence strategies in colonial Mesoamerica [10].

Yet current evidence of indigenous Mesoamerican animal management ranges from specialized breeding of scarlet macaw and turkey at Paquime (ca. A.D. 1200–1450) [11–13], to the ritual sacrifice of animals kept in Moctezuma’s zoo at the Aztec capital of Tenochitlan [1,14,15]. In fact, it is evident that Mesoamerican groups routinely captured, sequestered, and managed diverse species (e.g. perching birds, waterfowl, bees, deer, and rabbits) [10,16–20]. Among the Maya, a limited number of evidence including bird pens [21,22] and deer isotope values [20,23,24] suggest that animal management and trade was sometimes practiced beyond the two domesticates (i.e. dog and turkey). The rich zoomorphic iconographic tradition of the Maya documents how both animals and animal parts played a prominent role in human-animal dynamics beyond basic subsistence needs. Animals are depicted as gods, emblems of power, and as agents of influence over natural forces like water, sunlight, and darkness [25–27]. This study focuses on assemblages featuring human-animal interactions at the core of ritualized acts, often implemented to legitimize power in the domestic or public sphere. In particular, close attention is given to Mesoamerica’s premier emblems of rulership and power as the largest predators on the landscape, the jaguar (*Panthera onca*) [28–30] and the puma (*Puma concolor*) [31].

Stable isotope analysis enables researchers to document subtle changes in human-animal relationships by capturing shifts in diet, environmental alterations, and movement of humans and animals across the landscape. This study tracks differences in the stable isotopes of bone and teeth of six species excavated from ritual deposits at Copan to document the effective use and manipulation of wild carnivores at this important Maya center. A subset of the samples indicated anthropogenic diets documented in carbon isotopic values signaling high C_4_-based foodstuff (likely maize), while oxygen isotopic variation suggest that some of the fauna found in these ritual deposits were traded across the dynamic Maya landscape.

## Situating Copan and the zooarchaeological assemblage

**Fig 1:**
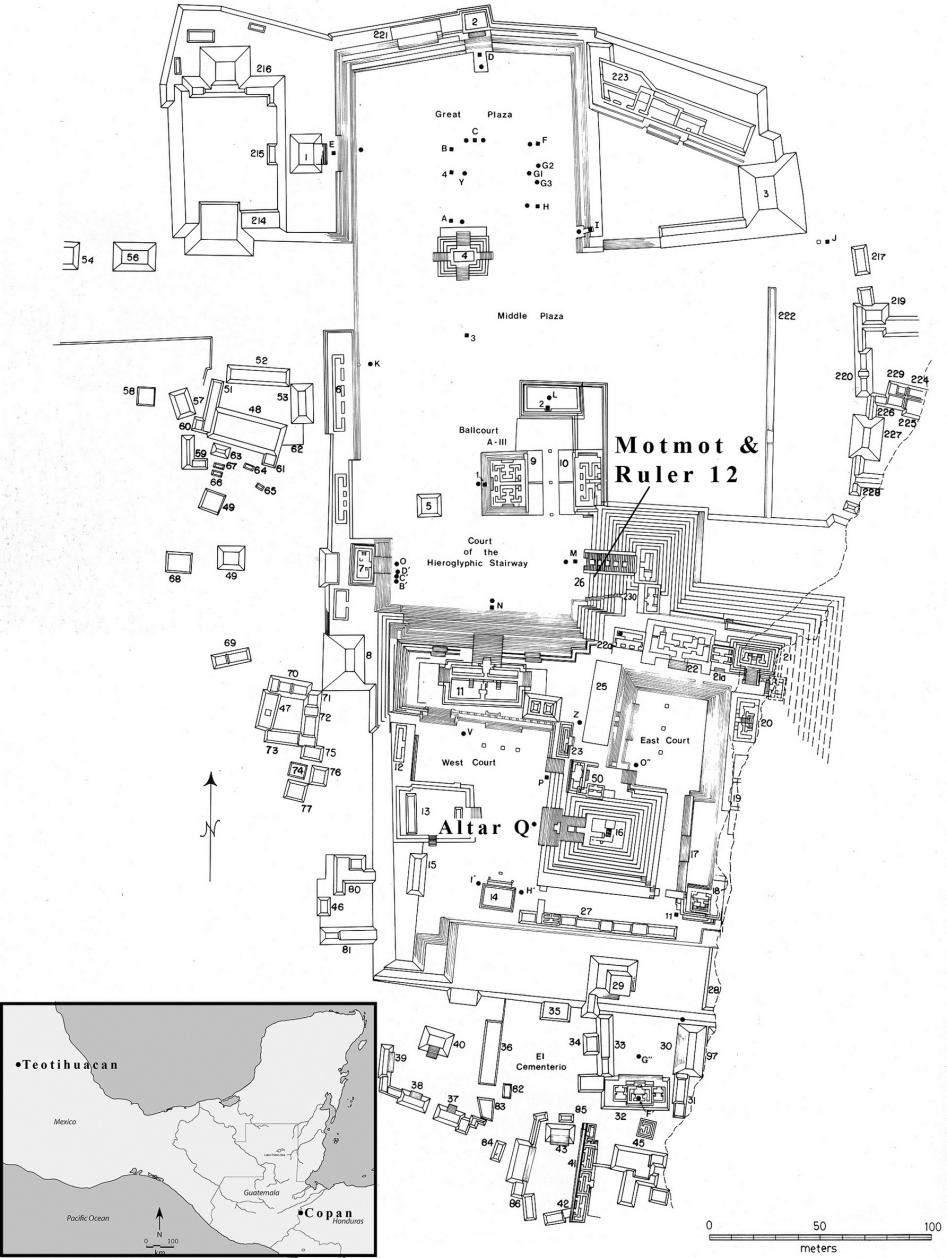
Map of the Principal Group at Copan highlighting location where Altar Q, Motmot burial, and Ruler 12 burial were excavated (modified from Fash 1991:Fig 8 under a CC BY license, with permission from Fash, original copyright 1991) and inset map of Mesoamerica highlighting location of Copan and Teotihuacan.

The Classic Maya city of Copan, located just east of the Guatemalan border in Honduras, was the premier site in the southeastern frontier of Mesoamerica (Fig 1). Elaborate sketches and descriptions by Stephens and Catherwood [32] drew international attention to this Maya center in 1841, spurring over a century of scientific fascination and increasingly rigorous investigation. Multiple archaeological expeditions document the historical trajectory of both the ceremonial compound, with its elaborate monumental and sculptural tradition, as well as several key residential sites in the urban core and hinterlands enclosed within the instrument-mapped 24 km^2^ extent of the city [33]. Copan was embedded within an expansive network of population centers linked by political and military alignments, trade flows, and cross-migration with other Maya settlements as well as distant Mesoamerican sites as far northward as the Central Mexican city of Teotihuacan [34–37]. Monumental inscriptions such as the longest contiguous Maya text known—the famous Hieroglyphic Stairway—record the dynastic lineage and key historical accomplishments of each ruler [38,39].

Both textual and archaeological evidence attest that fundamental connection with Teotihuacan was manifest from the inception of the Copan dynasty. Inscriptions recounting the journey of K’inich Yax K’uk’ Mo’, the city’s founding ruler, to Teotihuacan likewise express the influence of this distant center on the establishment of the royal lineage [40]. Many subsequent Copan rulers would continue to embrace Teotihuacan culture by mirroring its burial customs, architectural and iconographic styles, and by importing green obsidian from its local quarries [41]. Archaeological evidence demonstrates that the founder was buried in a building with a *talud-tablero* architectural style nearly identical to those of Teotihuacan [42]. In addition to green obsidian, numerous ceramic vessels traded out of Teotihuacan were identified in several royal burials in Copan, indicating their value as status enhancing ritual objects. Given this history, it is plausible K’inich Yax K’uk’ Mo’ and other Copan dignitaries witnessed how the Teotihuacan people captured wild carnivores and kept them in captivity in order to utilize them in state rituals at Teotihuacan.

Five assemblages spanning the Copan dynasty are considered, beginning with a cache placed during the transition to rulership from the founder to his son on A.D. 435 and ending with an offering placed during the reign of the sixteenth and final ruler on A.D. 776 next to Altar Q (Table 1). While the majority of the deposits examined originate from the ceremonial center, an elite residence and a control from residential middens across two status groups provide us with both a diachronic and synchronic framework of human-animal interactions at Copan. N. Sugiyama analyzed a total minimum number of 67 individuals (MNI 67) (exceptions are cited in Table 1), of which a subsample were integrated into the current isotopic investigation. As detailed description of the zooarchaeological study is forthcoming [43], here we simply summarize the zooarchaeological finds to contextualize the isotope results.

**Table 1:** Summary of zooarchaeological finds from this study.

Context/*Species*	Common name	Elements	MNI	Description
**Motmot Burial (A.D. 435)**				
*Mammals*				
*Puma concolor*	Puma	Complete	1	Complete, male between 2–3 years
*Odocoileus virginianus*	White-tailed deer	1 complete, 1 antler	2	Head analyzed, postcranial bones not available. Antler in Vessel 6
Rodentia	Rodent	Incomplete	1	Cranium, non-identified long bones
*Birds*				
Accipitridae	Small hawk	Incomplete	1	Head, shoulder, tail, and wings
*Corvus* sp.	Crow	7 phalanges	1	Phalanges
*Meleagris cf*. *gallopavo*	Domestic (?) turkey	1 Mostly complete, 1 atlas	2	One complete in Vessel 1 and one incomplete (atlas) in fill
Passeriformes	Small perching birds	Incomplete	2	Long bone elements and part of skull
Strigiformes	Owl	Semi-complete	1	Cranium, mandible, tarsometatarsus and some wing elements, Vessel 3
Aves	Non-ID bird	Incomplete	1	Small beak fragment, end of tarsometatarsus, both small birds
*Amphibian/reptile*				
*Crocodylus* sp.	Crocodile	Scutes	1	Not available for analyses, but documented during excavation.
Testudines	Small turtle	Various	2	At least one in Vessel 8, and a few more from general contexts
**Shaman's Burial (A.D. 450±50)**				
Mammalia	Unidentified mammal	Worked bone	1	Bone needle
*Odocoileus virginianus*	White-tailed deer	38 mandibles	19	Mandibles missing erupted teeth
*Crocodylus acutus*	American crocodile	Teeth	1	33 isolated teeth
Myliobatoidei	Stingray	Stingray spine	5	Spine covered with pigments
Testudines	Turtles	Carapace	2	Incomplete, many long bones, pending more analyses
**Ruler 12 (A.D. 695)**				
Felidae	Felid (puma/jaguar)	17 3rd and 2nd phalanx	2	Two pelts based on the distribution of the phalanges
**Altar Q (A.D. 776)**				
*Puma concolor*	Puma	Semi-complete	5	Mixed deposit
*Panthera onca*	Jaguar	Semi-complete	4	Mixed deposit
Felidae small	Small felid	Humerus	1	One humerus
Felidae large	Large felid (puma/jaguar)	Semi-complete	6	Mixed deposit
*Platalea ajaja*	Roseate spoonbill	Isolated long bones	1	Femur, humerus, and tarsometatarsus
*Ara* sp.	Macaw	9 bones	1	Not available for analyses, described by Ballinger and Stomper (2000)
**Midden contexts**				
*Odocoileus virginianus*	White-tailed deer	Ulna, scapula, metacarpal	4	See Collins (2002) for zooarchaeological analysis, only 4 sampled for isotopes
***Total MNI***			***67***	

### Motmot assemblage

The earliest faunal assemblage examined was recovered from the Principal Group, the ceremonial center of Copan. The zooarchaeological remains were found in a cylindrical masonry cist under the Motmot floor marker whose text and imagery document the succession from the founding ruler, K’inich Yax K’uk’ Mo’, to his son on the date 9.0.0.0.0 (A.D. 435) [35,36]. In this stone-lined cist, 1m wide and 1m deep, a young adult woman was buried cross-legged on a reed mat; her skeletal remains were accompanied by many offerings including eight complete ceramic vessels, shell, and lithic artifacts. In addition, three adult human skulls and a few neck vertebrae, a complete puma skeleton, and a deer skull were found intermixed with her bones [39,44]. This burial was later revisited, at which point the upper skeleton was disturbed and blackened by fire. The woman, interpreted to be an accomplished day-keeper, was entombed with the puma, likely her spiritual co-essence or *way* [45]. Other faunal remains were also scattered around the offering and placed inside a series of ceramic vessels interred with the body, possibly to serve as food for the afterlife.

The puma was found complete with no pathological indicators of captivity nor cause of death, and was in relatively good health at the time of death at the age of 2–3 years. An MNI of two white-tailed deer (*Odocoileus virginianus*) were also interred in the cache. The first individual was represented by a skull identified as that of a male 1.5 to 2.5 years of age. Unfortunately, the deer postcranial bones (documented by excavators *in situ* on top of the capstones that sealed contents of the stone-lined cist) were not available for analysis to confirm if they were from the same individual. A pair of antlers inside a ceramic vessel represent a second adult male deer. The remains of crocodile scutes (identified by Randolph Widmer during excavations), also recorded on top of the capstones to the cist, were likewise unavailable for analysis [Fash, Pers. Comm. 2017]. In addition, several other species of birds and turtles were identified, the majority found within the ceramic vessels. These included two turkeys, one of which was relatively complete. Given the species involved and the intentional placement inside the vessel, some of these remains were likely offered as prepared food (see 45 for full zooarchaeological report).

As the earliest evidence of puma sacrifice at Copan, this assemblage provided a primary context for investigating the possibility of captivity early in the Copan dynasty. In addition, the diverse species sampled from this ritual assemblage in the ceremonial center establish an isotope baseline for domestic (turkey) and wild (owl and deer) diets.

### Shaman burial, Plaza A of Group 9N-8

An elaborate burial was performed around the same time during the first half of the 5^th^ century A.D. in Plaza A of Group 9N-8. The animal accoutrements accompanying this individual identified him as a possible shaman [35,36]. Grave goods consisted of many ceramic vessels, two with quartz stones (possibly used in divination), highly elaborate shell artifacts (including 110 spondylus shell beads), a deteriorated bark-paper book or codex, as well as several faunal artifacts [35]. Five stingray spines (utensils for auto-sacrifice), a bone needle, two complete turtle shells, and one large collar, possibly a necklace made of faunal materials, were also identified. Despite the apparent high status of the interred subject, bioarchaeological analysis of this man about 45 years of age revealed indications of enamel hypoplasia from childhood [35].

**Fig 2:**
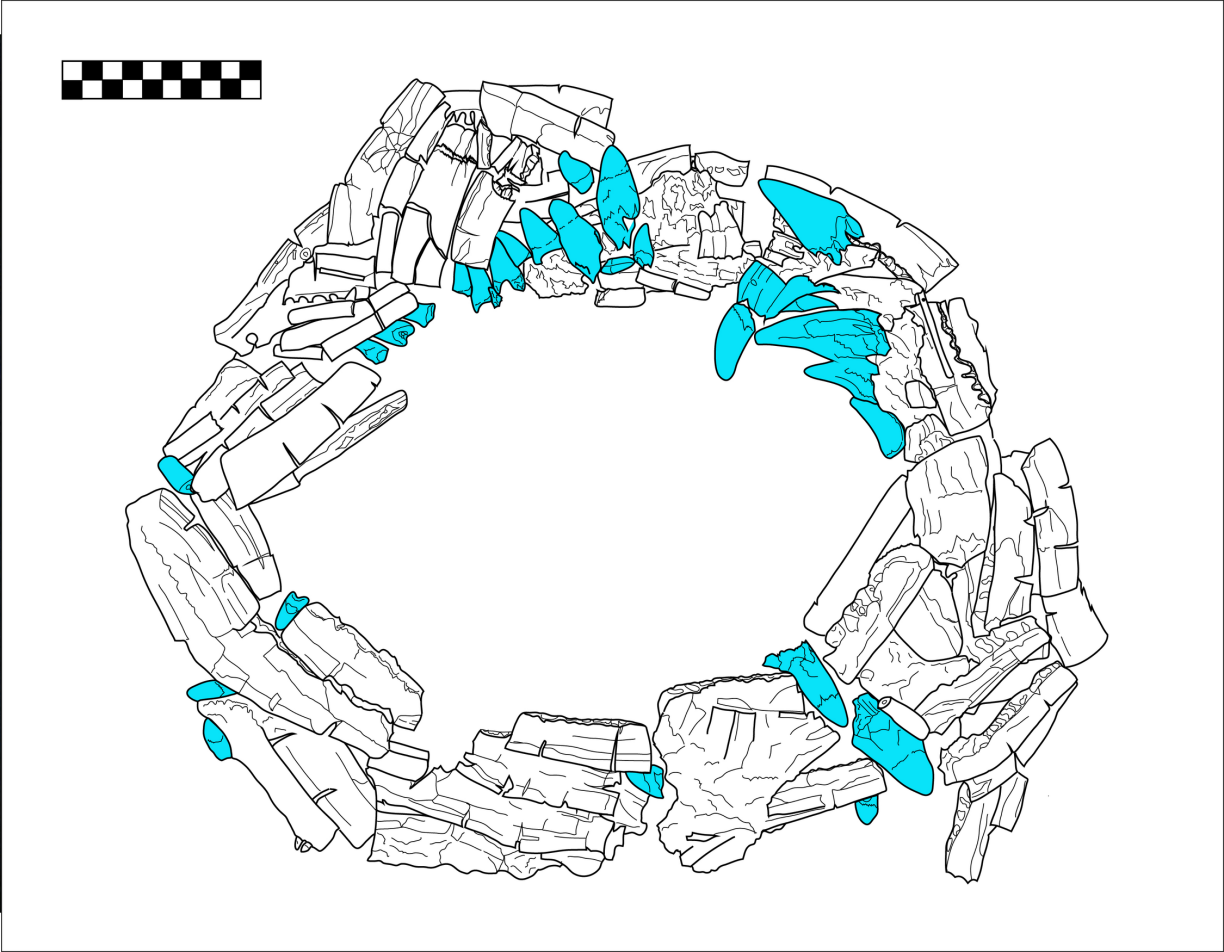
Plan view drawing of deer mandibles and crocodile teeth collar from Burial VIII-36, Plaza A of group 9N-8. Crocodile teeth are in blue. Drawing by N. Sugiyama.

The mandible collar is a circular arrangement of 38 mandibles totaling an MNI of 19 deer accentuated further by a group of 33 crocodilian teeth (observed in blue, Fig 2) accounting for an MNI of one crocodile. Most of the deer mandibles were modified by the carving of two large slits perpendicular to the mandible body, probably to facilitate tying these elements together. Some surfaces also showed evidence of polishing. Unfortunately, the consolidants applied at the time of excavation made additional analysis of surface features extremely difficult, especially because of the many crushed and disfigured elements.

Most likely the crocodilian teeth are of the American crocodile (*Crocodylus acutus*) and not the Morelet’s crocodile (*Crocodylus moreletii*) as it is only found in Atlantic and Caribbean lowlands of Mexico, Guatemala, and Belize [46,47]. One worked bone needle and a stingray spine was affixed to the bottom of the collar with consolidants, making this one of five stingray spines placed in the center of this feature. A sprinkle of red cinnabar dust was still discernable on the surfaces of both the stingray spines and the collar feature.

Deer mandibles included specimens from both young individuals with teeth still erupting in the sockets, to full adult mandibles lacking any dentition. All deer permanent dentition was deliberately extracted from the mandibles, while isolated crocodilian teeth were mixed into the assemblage. The crocodilian teeth were arranged to create a composite deer-crocodile hybrid feature. This artifact may be representing the so called “Cosmic Monster” [48] or “Starry Deer Crocodile” [49] found abundantly in Maya iconography and writing [43]. The use of crocodilian artifacts by non-royal elites is significant given the animal’s frequent motif as divine axis of the Maya cosmic order, often depicted as a crocodilian tree, or adorning royal individuals [48]. It is particularly noteworthy that the ancient Maya were not only drawing hybrid animals, but were physically manipulating bones and teeth of their constituent natural subjects to create tangible composite artifacts which directly materialize these cosmological beings.

This assemblage represents the ritual use of fauna by elites outside the Principal Group. The deer sampled from this necklace provide another data point for deer isotope composition at Copan, in this case from ritual regalia. Furthermore, the crocodile teeth analyzed mark the first Mesoamerican crocodile isotope data point, providing us with preliminary data that we hypothesize would be distinct from terrestrial foodwebs.

### Burial of ruler 12

The tomb of Ruler 12, K’ak’ Uti’ K’awil (A.D. 625–695), the longest ruling monarch of the Copan dynasty, contained one of the most elaborate burials. This included twelve human effigy censer lids depicting Ruler 12 and each of his predecessors. A well-preserved child was found in close proximity, but the ruler’s body had been wrapped in a cocoon of unfired clay which hindered bioarchaeological investigation because the soil’s acidity led to poor preservation [35]. Royal grave contents included 113 ceramic vessels, oyster shells, and jade artifacts, among other goods.

Faunal remains from Ruler 12’s burial were limited to 17 felid third phalanges (bone on which the cartilaginous claw attaches) and three second phalanges totaling an MNI of one. Often, the exclusive presence of third and second phalanges implies they are remnants of pelts, as these bones are difficult to separate from the skin. Excavators identified the presence of two pelts based on the distribution of these osseous remains [35]. Unfortunately, felid phalanges are not diagnostic to a species level and can only be characterized as a felid of jaguar or puma size. Iconographic and ethnographic evidence demonstrate a close connection between royal lineages and jaguar symbolism [28–30,50]. Jaguar pelt mats frequently symbolized seats of power throughout Mesoamerica, supporting the hypothesis that these phalanges most likely originate from jaguars.

Not only were animal bones and complete animals important in ritual, but their pelts and other secondary products were also highly prized items utilized extensively as symbols of dominance and power at the Principal Group. As the majority of the other felid samples are of primary sacrificial victims, the felid phalanges will define differences in isotope signatures between faunal products, in this case pelts, and primary burials. In chronological terms, Ruler 12’s tomb lies between the Motmot and Altar Q assemblages (A.D. 695), and thus represents an additional data point between these two periods.

### Altar Q

Materials from Altar Q furnish evidence of a state-sponsored mass sacrifice event at Copan with a striking resemblance to practices originating at Teotihuacan. In commemoration of the completion of Temple 16 (Building 10L-16), the sixteenth and last ruler of Copan, Yax Pasaj Chan Yopaat (reigned A.D. 763–820) commissioned the placement of Altar Q in front of this structure in A.D. 776. A stone masonry crypt immediately in front of the altar sealed a dense concentration of faunal remains that was excavated by Jeffrey Stomper in 1988. Original analysis by Ballinger and Stomper [51] identified a total MNI of 14 felids ― at least six of which are jaguars ― and nine avian specimens offered during the dedicatory ritual.

It is particularly pertinent to examine the faunal cache in context of the sociopolitical instability confronting this final ruler; a circumstance potentially exacerbated if not partially triggered by deforestation and environmental stress [52,53] (see however [54]). Altar Q can be interpreted as a display of domination. Etched in hieroglyphic text on the superior surface is the legendary voyage by which the founding king, K’inich Yax K’uk’ Mo, inaugurated the Copan dynasty. His 15 predecessors, arrayed along the four sides of the altar, successively relay the baton of royal authority to the final king, legitimizing Yax Pasaj’s place atop this storied hierarchy [55]. These statements are sometimes construed to evoke a glorification scene wherein the last ruler of Copan, Yax Pasaj, sacrifices ferocious jaguars above the altar in dedication to his dynastic ancestors.

Ballinger and Stomper [51] calculated a home range of 8 km^2^ for this highly territorial felid in order to argue that the Copan Valley, at 42 km^2^ large, could only support a locally available population of five individuals. The highest observed densities for jaguars in areas of extremely high prey availability is 10 to 11 km^2^ per individual, and normally extends between 25–38 km^2^ (females) to 50–75 km^2^ (males) [56,57]. It is thus difficult to hypothesize how Yax Pasaj was able to procure such a high quantity of these animals from the valley; especially given the adverse social, political, and environmental pressures at work.

Any or all of the following alternative and mutually compatible scenarios could resolve this conundrum. First is the incorporation of another type of felid, such as the puma, which is both equally abundant and often morphologically indistinguishable from the jaguar. Second is to keep a population of captive animals in order to obtain the necessary number of individuals for the ritual, a methodology observed at Teotihuacan [2]. Third is the secondary deposition of jaguar or puma skeletons. Finally, jaguars and pumas may have been traded from other regions. Based on these hypothetical scenarios, three primary goals were defined for analyzing Altar Q fauna: 1) total count (MNI) and species distribution of the felid assemblage; 2) identifying surface modifications to reconstruct taphonomic histories and pathological indicators of captivity; and 3) collecting samples for isotopic investigation.

Ascertaining the MNI was unusually challenging due to the mixed deposition observed during excavations. Felid elements were so densely aggregated that this assemblage was coined “jaguar stew” by the excavators [Fash, personal communication 2008]. A recalculation, based mainly on dentition, determined that 16 felids were present, rather than 14 as previously argued [51]. This calculation included the presence of four jaguars and five pumas, and even a humerus from one smaller felid of jaguarundi or ocelot size [43].

A few surface modifications including cutmarks, carnivore tooth marks, and rodent gnawing were recorded in addition to the differential weathering stages which indicate surface exposure [58]. Together, these modifications suggest at least some of the skeletons were cached secondarily alongside sacrificed felids. This explains the presence of articulated components within the otherwise irregular distribution of animal parts within the deposit. Rodent activity or other post-depositional disturbances do not adequately explain the differences observed between the expected patterns (complete skeletons) and the archaeological remains uncovered (a combination of weathered, fragmentary, and disarticulated remains).

The remains of a roseate spoonbill (*Platalea ajaja*) (femur, humerus, and tarsometatarsus bone) were also found. Excavation records also describe the remains of a macaw (*Ara macao*), although this was not available at time of analysis. The presence of the spoonbill, usually found in coastal habitats, is noteworthy given its non-local distribution. Both the macaw and the spoonbill were highly valued, as their elaborately colored plumage was often incorporated into ritual accoutrements.

The faunal materials from Altar Q appear to furnish the only evidence of a state-sponsored animal sacrifice event at Copan of comparable scale to practices originating at Teotihuacan. This assemblage dates to the last evidence of state-sponsored ritual sacrifice at Copan prior to its collapse, providing a good marker to compare to earlier deposits like the Motmot assemblage for continuity or change. The spoonbill specimen further enhances the species diversity in this study.

### Control, midden contexts

As a control, four deer specimens from two middens were also analyzed for their isotopic composition based on the identification provided by Collins [59]. In particular, cervid bones from OP15 and 17 were sampled, avoiding materials from Level 1. These were selected because according to Collins [59], OP15 was an intermediately ranked residence while OP17 was a low status residence. These deer specimens represent domestic consumption at two residences in the Copan Valley.

These varied contexts selected from Copan exemplify the multiple ways in which animals were a fundamental component of ritualized acts, both at the ceremonial center and in elite residences. Baselines and controls for a variety of wild and domestic species that span the Copan dynasty were taken. Re-analysis of some of the best evidence of animal sacrifice directly tied to the Copan state at Altar Q captured the unique socio-political struggles faced by Copan’s regime, while at the same time demonstrated the extent to which animals were manipulated and mobilized across the landscape. More detailed descriptions of the zooarchaeological study of Altar Q, Motmot, and the shaman burial from Plaza A of Group 9N-8 can be found elsewhere [43].

## Stable isotopes

Stable isotope analyses of bone and teeth were implemented to examine the diet and geographical origin of the animals. All isotope compositions are given in per mil (‰) units and standard delta notation is utilized for reported isotope values derived from the equation,

$$\delta =\left[\left(R_{sample}–R_{standard}\right)/\left(R_{standard}\right)\right]*1000$$

where R = ^13^C/^12^C, ^15^N/^14^N, or ^18^O/^16^O. The standards used were V-PDB for C, atmospheric air for N, and V-SMOW for O. Each tooth and bone consisted of organic (collagen) and mineral (carbonate) components. Bone and tooth dentine were analyzed to obtain simultaneous δ^13^C_collagen_ and δ^15^N_collagen_. Tooth enamel, tooth dentine, and bone were sources for δ^13^C_carbonate_ and δ^18^O_carbonate_ data.

Differences in carbon isotope ratios, expressed as δ^13^C, reflect variations in the relative inputs of different plant resources, distinguished by their photosynthetic pathways. Most trees, shrubs, and temperate grasses are signaled by Calvin cycle (C_3_) isotope ratios that range around -26.5‰. The Hatch-Slack Cycle (C_4_) is typically found in arid to sub-arid regions and ranges around -12.5‰. Values for Crassulacean Acid Metabolism (CAM), common to epiphytes and xerophytes living in hot and arid environments, span the C_3_ and C_4_ ranges but overlap more substantially with C_4_ values [60,61]. In the tropical habitats of Honduras, where C3 grasses dominate the environment, the most prevalent C_4_ crop would have been agricultural maize (*Zea mays*).

Carbon isotope ratios can be obtained from both the mineral apatite-carbonate (δ^13^C_carbonate_) and organic collagen (δ^13^C_collagen_) phases, each yielding slightly different information. The former provides a value of the total diet, while the latter is biased toward dietary protein [62–66]. Combining carbon isotope data from collagen and carbonate phases enables researchers to model the source of C_3_ or C_4_/marine values (protein vs. total diet) with consistent results across different body sizes and physiological processes [63,64]. Kellner and Schoeninger [64] validated their application by plotting well-characterized human populations against a pair of parallel regression lines generated by various experimental studies on rats, mice, and pigs of known C_3_ or C_4_ diets. This simple carbon model has proved to be an effective instrument for visualizing the δ^13^C values from multiple dietary sources across different populations [65,67]. By analyzing δ^13^C of mineral apatite-carbonate and collagen, this study will generate a simple carbon model, allowing us to distinguish between C_3_ or C_4_/marine sources.

Nitrogen stable isotope ratios in collagen usually reflects the trophic level of the consuming organism. While other factors such as drought and agricultural practices can affect the baseline values of the plants [68–70], generally speaking, elevated δ^15^N_collagen_ values correspond to higher trophic levels, with a 3–5‰ enrichment at each level [71,72]. In addition, because marine ecosystems have a greater number of trophic levels, δ^15^N_collagen_ values of marine resources are enriched by 6–8‰, allowing us to measure the proportion of terrestrial versus marine food sources in the organism’s diet [73,74]. Taken together, nitrogen and carbon isotope composition can indicate the origin of a particular plant resource as well as distinguish between direct consumption (e.g. jaguar eating corn) and indirect consumption (e.g. jaguar eating turkey eating corn).

Carbon and nitrogen isotope composition, both separately and in combined protocols, have been utilized to identify instances of New World animal captivity. Human dietary intervention results in diets substantially altered from wild controls, reflected in correspondingly distinct animal isotope compositions. For example, macaws penned at the pre-Hispanic site of Paquimé in northern Mexico [12], turkeys in the ancient Southwestern United States and in the Maya region [8,75], or lagomorphs kept in captivity in Teotihuacan [18,19] were identified isotopically because of their conspicuously elevated or even total reliance on C_4_ based resources. Among domesticated dogs of the Maya, this increased reliance on C_4_ resources correlated with a decrease in nitrogen isotope ratios, an indication that meat protein input decreased as reliance on agricultural maize increased [20].

More recently, animals sacrificed in the dedicatory chambers at the Moon Pyramid and Sun Pyramid were determined to have been extensively managed based on observed high input of C_4_ foodstuffs [2]. In contrast, animals interred as secondary products exhibited the relatively low C_4_ inputs diagnostic of a “wild” diet. We would likewise expect to see similar bimodality at Copan, with one cluster consisting of animals hunted from a wild population with high C_3_ plant intake, and the other of animals raised in captivity which possess a distinctly elevated C_4_ composition (and thus a more positive δ^13^C value); the expected result of a diet incorporating C_4_ consuming herbivores (retaining high δ^15^N values).

Oxygen isotope ratios, expressed as *δ*^18^O_carbonate_, can be read from bone, tooth enamel, and tooth dentine. As the *δ*^18^O values varies by the water source an organism drinks (which in turn reflects the climate and geography of the region) and the organism’s physiology, oxygen isotope ratios can illuminate origin and migration patterns of humans and animals [8,23,76–78]. Throughout Mesoamerica, extensive work on oxygen and strontium isotopes on human skeletal remains have helped develop isoscapes of the regions studied [79–83]. Wassenaar [84] has created a map of groundwater *δ*^18^O variation across Mexico, ranging between -10‰ and -4‰, with more negative values distributed in higher elevations (Central Highlands), while more positive values are found in lower elevations (Yucatan peninsula and Gulf Coast of Mexico). Human skeletal remains from Copan defined local ranges between -3.2‰ to -5.6‰ (±1s.d.), with more *δ*^18^O variation identified between sites than chronological shifts during the Copan occupation [85,86]. Because body physiology (size and behavioral patterns) affects oxygen isotope ratios, isoscapes modelled on human isotope values cannot be utilized to plot cross-species isotopic results from the various fauna of Copan onto a map. δ^18^O values are also influenced by reservoir effects, where still water in lakes, ponds, and storage vessels exhibit higher δ^18^O values due to evaporation of the lighter isotope. In addition, cautionary evidence of water sources from the Maya region illustrated local *δ*^18^O variation can be quite extensive [87]. However, as the human data has demonstrated, variation in *δ*^18^O values within a species may correspond with different geographic origins of individual animals in a population, even though these values do not directly identify distinct regions.

**Fig 3:**
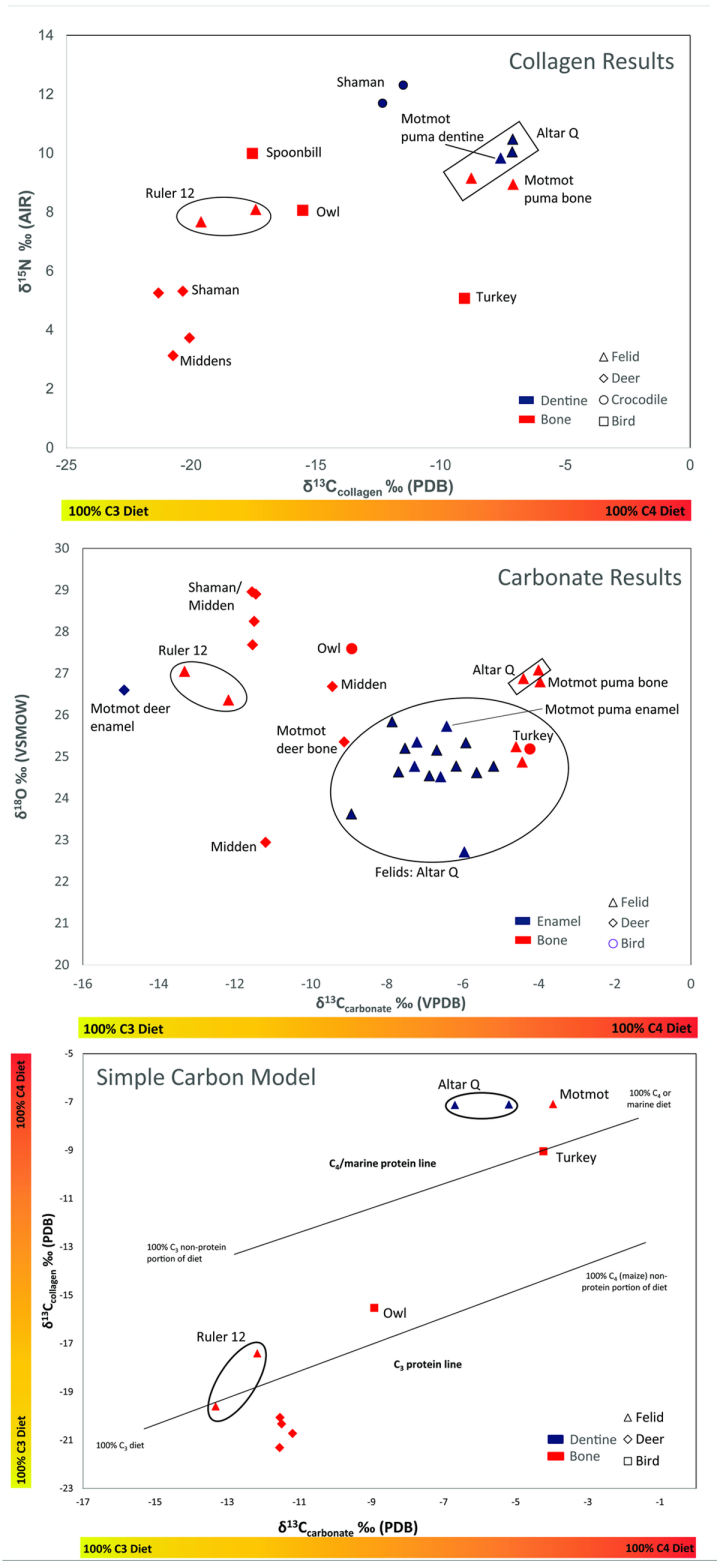
Results of stable isotope analysis by animal type: a) δ^13^C_collagen_ and δ^15^N_collagen_ values, b) δ^13^C_carbonate_ and δ^18^O_carbonate_ values, and c) simple carbon model following Kellner and Schoeninger [64].

Differences between bone, tooth enamel, and tooth dentine isotope values record changes in subsistence and migration patterns that occur between the individual’s youth (enamel) and lifetime average of adulthood (bone and tooth dentine). This is because enamel mineralizes once, capturing the short span of tooth formation, while bone and tooth dentine remodels constantly throughout the lifetime of the organism, capturing the average isotopic input during its adult life; this capture window varies in length depending on the species and element analyzed [88,89]. Enamel is less susceptible to diagenesis, yet can introduce variability due to breastfed and weaned diets, as well as seasonal and annual fluctuations. Dentine was used only when no other values were available to represent the individual because preservation is a major factor of concern. No dentine carbonates were considered, and only five dentine collagen samples that passed the diagenesis tests are included. The mostly disarticulated context permitted obtaining bone and tooth from the same individual on only three animals. Of these, samples from two individuals passed the diagenesis test: a complete articulated puma from the Motmot burial, and a deer skull from the same context. These two individuals are labeled in Fig 3.

### Samples

A total of 54 samples were taken from each of the five zooarchaeological assemblages described above and analyzed for carbon, nitrogen, and oxygen stable isotope composition (Table 2). Teeth and bone from the Motmot burial’s puma (M1, I3, and second metatarsal) and deer (cranial fragment, left lower 4^th^ premolar) were selected to distinguish between isotope values during infancy (dental enamel) and adulthood (bone). In addition, as diagenesis affects bone, teeth dentine, and teeth enamel differently, taking all available data types for each individual could result in better sample sizes. Avian species (turkey, owl, and spoonbill), lacking dentition, naturally only produce bone specimens. From the shaman burial in Plaza A of Group 9N-8, two crocodile teeth and two mandibular deer fragments were selected for analysis. Three felid phalanges from Ruler 12’s tomb were also included in the study. A fragment of the left proximal radius (*n* = 13) and the left upper fourth premolar (*n* = 13) were sampled from the felids in Altar Q to obtain maximum representation while discounting duplication within this highly mixed deposit. In the latter case, this tooth was used to conduct species-level identification, allowing for jaguar and puma isotope values to be compared despite many isolated teeth. Two cranial fragments were also taken, but did not pass the diagenesis test. In addition, the humerus of a smaller felid (neither jaguar nor puma), along with two left inferior first molars and two mandible fragments of jaguar were extracted. Deer specimens from mixed midden deposits were taken as a control (*n* = 4). These isolated elements were sourced from different strata to ensure the same individual was not re-sampled.

**Table 2:** Number of elements sampled from each context for isotope analysis.

	*Element*	*N*
**Motmot**		
Puma	Teeth: M1 and I3	2
	Metatarsus II (L)	1
Deer	Cranium	1
	Teeth Pm4 inf (L)	1
Turkey	Humerus (L)	1
Owl	Ulna (R)	1
Spoonbill	Femur (L)	1
	Humerus (L)	1
	Tarsometatarsus	1
*Total*		*10*
**Shaman Burial, Plaza A of Group 9N-8**		
Crocodile	Teeth	2
Deer	Mandible	2
*Total*		*4*
**Ruler 12**		
Felidae	Phalanx	3
**Altar Q**		
Felidae	Teeth Pm4 sup (L)	11
	Radius (R)	13
	Humerus	1
Jaguar	Teeth Pm4 sup (L)	2
	Teeth M1 inf (L)	2
	Mandible/cranium	4
*Total*		*33*
**Midden**		
Deer	Ulna (L)	2
	Scapula	1
	Metacarpal	1
*Total*		*4*
***Grand Total***		***54***

R = right, L = left, M = molar, Pm = premolar, I = incisor.

### Methodology

When necessary, bone and teeth were cut with a diamond tip drill in preparation for destructive analysis. These elements were exported with permission from the Honduran Institute of Anthropology and History (Authorization 005–2014) and analyzed at the Smithsonian MCI Stable Isotope Mass Spectrometry Laboratory. Both collagen and structural carbonate were extracted for their respective analyses.

Collagen extraction protocol followed standard methods per Longin [90] and modified per France et al. [91]. Solid bone and (when available) dentine fragments (~ 200–800 mg) were sonicated in ultrapure water to remove any surface sediments and labile salts. With the exception of the samples from the shaman burial necklace, the application of consolidants was not noted at time of examination and the surface cleaning applied was deemed sufficient for isotope analysis. Four samples bearing consolidant residue (most likely diluted conventional glue), and one element with an excessively eroded surface (NS38) required additional cleaning steps. These included two overnight acetone soaks, three ultrasonic washes in acetone, and five ultrasonic washes in ultra-pure H_2_O (18.2 MΩ-cm).

Once cleaned and weighed, samples were demineralized with 0.6M hydrochloric acid (HCl) in 4°C conditions, changing the solution every 24 hours until reactions ceased (average 2–7 days). Samples were rinsed to neutrality in ultrapure water, and humic acids were removed by soaking in 0.125M sodium hydroxide (NaOH) for 24 hours at room temperature. Samples were rinsed to neutrality and the remaining organic material was solubilized by an overnight soak in 0.03M HCl at 95°C to separate soluble and insoluble phases of collagen. The soluble phases of collagen were isolated and lyophilized to produce a purified collagen extract. These samples were subsequently weighed into tin cups and combusted in a Costech 4010 Elemental Analyzer coupled to a Thermo Delta V Advantage mass spectrometer. The *δ*^13^C_collagen_, *δ*^15^N_collagen_ values are calibrated against an internal acetanilide and urea_UIN3 reference materials, both calibrated to USGS40 and USGS41[92]. Error is ±0.2‰ (1σ) for both carbon and nitrogen values.

Structural carbonate in the mineral apatite was extracted from bones, tooth dentine, and tooth enamel according to modified methods of Bryant et al. [93]. Approximately 20-30mg of bone or tooth was removed and ground into a fine powder using an agate mortar and pestle, then soaked overnight in 2–3% sodium hypochlorite (NaClO) to remove organic components. Samples were rinsed with ultrapure water, then reacted with 1M acetic acid solution buffered with 1M calcium acetate (pH~4.5) for 4 hours to remove secondary carbonate phases [94,95]. Once samples were rinsed to neutrality and dried, they were weighed into Exetainer vials, reacted with concentrated (>1.92 SG) phosphoric acid (H_3_PO_4_), and analyzed on a Thermo Gas Bench II connected to a Thermo Delta V Advantage mass spectrometer. The *δ*^13^C_carbonate_, *δ*^18^O_carbonate_, values were calibrated against NBS-19 and LSVEC carbonate reference materials and have an error of ±0.2‰ (1σ).

Diagenesis on collagen samples was assessed according to previously determined criteria for collagen (% collagen yield greater than 1%, C/N ratio of 2.8–3.6, and % N yield of 11–16%) following pertinent literature [96,97].

Degree of diagenesis in bone carbonate samples was assessed utilizing the Fourier-Transform Infrared Spectrometer with the attenuated total reflectance (FTIR-ATR) attachment at the Smithsonian Museum Conservation Institute. Two criteria were utilized: C/P ratios (mineral carbonate phosphate) and IR-SF (mineral crystallinity index) values. C/P ratios are constituted by the ratio of the carbonate and phosphate peaks at 1415 cm^-1^ and 1035 cm^-1^ on the FTIR spectrum [98,99]. Modern bone averages approximately 0.5 and values below 0.1 were omitted as they signify the presence of extensively degraded minerals [98–100]. IR-SF values, calculated as [565_ht_+605_ht_]/590_ht_, indicate the splitting factor of the phosphate peaks [99,101]. Values above 4.0 usually indicate degraded or burnt bone and have been excluded from further analysis.

The three sources of data (bone, tooth dentine, and tooth enamel) are distinguished in the Fig 3. As enamel apatite tends to be enriched over bone by 2.3 ‰ in carbon and 1.7 ‰ in oxygen [102,103], tooth isotope ratios were corrected to match bone in all Fig 3 and Table 3 (Note: Supplementary Data report raw data prior to corrections).

**Table 3:** Descriptive statistics of the isotope values that passed diagenesis testing by animal type.

		δ^13^C_collagen_ (‰ VPDB)	δ^15^N_collagen_ (‰ AIR)	δ^13^C_carbonate_ (‰ VPDB)	δ^18^O_carbonate_ (‰ VSMOW)
*Felid*					
	*N*	7	7	22*	22*
	Mean	-10.7	9.2	-6.8	25.3
	St Dev	5.4	1.0	2.4	1.1
	Low	-19.6	7.7	-13.3	22.7
	High	-7.1	10.5	-3.7	27.4
*Deer*					
	*N*	4	4	8*	8*
	Mean	-20.6	4.4	-11.3	26.9
	St Dev	0.5	1.1	1.7	2.0
	Low	-21.3	3.1	-14.9	22.9
	High	-20.1	5.3	-9.1	29.0
*Bird*					
	*N*	3	3	2	2
	Mean	-14.0	7.7	-6.6	26.4
	St Dev	4.4	2.5	3.3	1.7
	Low	-17.5	5.1	-8.9	25.2
	High	-9.0	10.0	-4.2	27.6
*Crocodile*					
	*N*	2	2	-	-
	Mean	-11.9	12.0	-	-
	St Dev	0.6	0.4	-	-
	Low	-12.3	11.7	-	-
	High	-11.5	12.3	-	-
***Total***					
	*N*	16	16	32	32
	Mean	-13.9	8.0	-7.8	25.9
	St Dev	5.6	2.8	3.1	1.5
	Low	-21.3	3.1	-14.9	22.9
	High	-7.1	12.3	-3.7	29.0

*Includes a tooth and a bone sample from the same individual.

## Results and discussion

Descriptive statistics of the entire assemblage are summarized in Table 3, and raw data can be accessed in the supplementary S1 Table. Preservation of collagen samples was very poor. Fifty-four samples of bone and teeth dentine were processed for collagen extraction, of which only 16 passed the diagenesis criteria across contexts and species, accounting for 30% of the total (dentine *n* = 5/20, bone *n* = 11/34).

Felid δ^13^C_collagen_ data exhibit a bimodal distribution that cannot be explained by chronological differences (Fig 3A). Two felid claws in Ruler 12’s tomb (A.D. 695) average -18.5‰, a value that falls within the expected range for a C_3_ dominated landscape, as do the four deer, the spoonbill, and the owl specimens which range between -15.5 to -20.1‰. In contrast, the puma from the Motmot burial (A.D. 435) and three felid samples from Altar Q (A.D. 776) have unnaturally high C_4_-based diets. In fact, their isotope values suggest the felids were consuming more C_4_ (maize) based resources (average -7.5‰) than the domesticated turkey (-9.0‰) found in the Motmot burial. Although less pronounced than δ^15^N_collagen_ values, δ^13^C_collagen_ do exhibit a roughly 1.2‰ trophic level effect within a single foodweb [104], which may be reflected in this case between the consumer (felid) and its source (turkey).

Motmot and Altar Q felids likely represent instances of primary animal sacrifice. In contrast, the phalanges analyzed from Ruler 12’s burial chamber were most likely secondary by-products of pelts and these two δ^13^C_collagen_ values are significantly lower (*p* < 0.001) than the rest of the felidae population. The isotope values sourced from the Altar Q and Motmot felids are comparable to those of primary burial felids identified at Teotihucan, with C_4_ signatures extending as high as -6.2‰ [2]. This data strongly supports the hypothesis that these four felids were fed artificial diets. The fact that both Motmot (A.D. 435) and Altar Q (A.D. 775) felid samples illustrate evidence of captivity suggests long-term continuity in the practice throughout the Copan dynasty.

As expected, all felid populations have more enriched δ^15^N_collagen_ values (about one to two trophic levels higher, $\overset{-}{x}$ = 9.2‰) than the deer ($\overset{-}{x}$ = 4.4‰) and the turkey (5.1‰), indicating that C_4_ resources entered their diet though indirect pathways (C_4_-fed prey). The spoonbill and the owl have the expected C_3_-based diets (δ^13^C_collagen_ = -17.5 and -15.5‰ respectively) with enriched δ^15^N values (+10.0 and +8.1‰ respectively) correlated with natural resources. The riverine source of the crocodile’s diet could explain both the high δ^15^N_collagen_ values and the presence of a mixed C_3_ and C_4_ diet, distinct from the terrestrial habitat, although a more robust sample size is necessary to form any conclusion.

Out of the 73 samples processed for structural carbonate, 36 passed diagenesis testing accounting for 49% of the total (dentine *n* = 3/20, enamel *n* = 17/19, bone *n* = 16/34), providing a more robust sample (*n* = 32, excluding dentine samples and one duplicate enamel sample) (Fig 3B and Table 3). Schwarcz’s [105] formula calculates the percentage of contribution of C_4_ based products from carbon apatite isotope values:

$$\%C_{4}=(\left(\delta_{3-}\left(\delta_{b}-\Delta \right)/\delta_{4}–\delta_{3}\right)*100$$

where δ_3_ is the end member δ^13^C value for C3 foods (-25‰) and δ_4_ is the assumed end member δ^13^C value for C_4_ goods; in this case most likely archaeological maize (-9‰). δ_b_ is the measured δ^13^C_carbonate_ value of Copan samples, and Δ is the δ^13^C_carbonate-diet_ spacing (9.7‰) [64,105].

Applied to the low and high values of the entire assemblage, % C_4_ intake for all carbonate samples was between 3 and 73%. The felid samples extended through this entire range, clustered into two distinct populations. According to a two-tailed t-test, the two specimens from Ruler 12 have a significantly low C_4_ intake ($\overset{-}{x}$ =-12.7‰, 16% C_4_), while the rest of the population from Motmot and Altar Q deposits was higher ($\overset{-}{x}$ = -5.9‰, 59% C_4_) (*p* < 0.001). Some individuals demonstrate an abnormally high reliance on C_4_ resources: δ^13^C_carbonate_ values of three felids were more positive than a domestic turkey (-4.2‰, 69% C_4_), suggesting this may be their protein source. These values are as high as some of the felids analyzed from Teotihuacan; a group of animals with clear pathological indicators of captivity (average complete skeletons -6.2‰, 57% C_4_) [2].

Deer naturally congregate around milpa plots, not only to consume agricultural products, but also to lick the salts generated from slash-and-burn agricultural practices. Indeed, garden hunting is documented as an effective strategy among tropical groups [106]. This may explain why C_4_ resources seem to have played a role among the control deer carbonate samples ($\overset{-}{x}$ = -11.3‰, 25% C_4_). The owl also exhibits a somewhat elevated C_4_ intake (-8.9‰, 40% C4), which may hint at the degree of landscape alteration caused by the introduction of C_4_ grasses into the area.

Based on the collagen and carbonate results, a simple carbon model was developed in order to determine whether the source of the high C_4_ intake was overall dietary contribution or protein specific (Fig 3C). As this requires both collagen and carbonate results for the same bone/tooth in question, eligibility constrained the number of samples to be plotted (*n* = 11). The observations made in collagen and carbonate values are accentuated in this model. There is a strong association of felids from Ruler 12, closely aligned along the C_3_ protein line, closer to the 100% C_3_ diet range. Altar Q and Motmot felids, on the other hand, are clearly above the C_4_/marine protein line, closer to the limit of the 100% C_4_/marine diet range, at the opposite end of the spectrum. Given that the turkey is found in a very similar location along the C_4_/marine protein line as the latter, it is likely the felids were fed these domesticated animals. Deer, on the other hand, closely cluster near the two felid values from Ruler 12’s tomb, suggestive of a wild signature. As before, the owl displays an intermediate range.

While the inter-species differences in oxygen values cannot be used to directly plot movement of animals across the landscape, intra-species variation can signal unusual or abnormal relocation patterns. Deer had the largest variation in δ^18^O_carbonate_ values (Table 3, 6‰ range). Given that groundwater isoscape models developed by Wassenaar et al. [84] exhibited 6‰ variation over the entirety of Mexico, the observed deer isotope variation is large for such a comparative small region. A single deer sample fell outside the one standard deviation range at +22.9‰. This low number suggests it originated in a more arid climate, although multiple factors including elevation, proximity to the ocean, etc. need to be considered. The δ^18^O_carbonate_ values of a deer tooth and bone from the Motmot burial chamber suggests this deer changed its habitat and/or diet during its lifespan. The tooth sample (NS35e, 28.3‰) was 2.95‰ more enriched than its bone (NS34, 25.35‰). While the limited deer sample size is insufficient to accurately document the local signature, this distribution is in agreement with other [23,24] oxygen and strontium isotope study on deer, peccary, and dog skeletons throughout several Maya sites that identified long-distance exchange networks transporting faunal products across the dynamic Maya landscape. Additional strontium isotope work at Copan could help further define the extent and range of faunal transport across Mesoamerica.

The δ^18^O_carbonate_ variations observed among jaguars and puma samples are more challenging to interpret. As both jaguars and pumas have extensive home ranges, and are quite intolerant of territorial overlap (jaguars 25-38km^2^, pumas 90-300km^2^) [56,107], the extensive δ^18^O_carbonate_ variation is not surprising. However, 4‰ variation is high, with some samples clustering above one standard deviation from the mean (+25.2‰). This group consists of two claws from Ruler 12’s tomb (+27.0 and +26.0 ‰) and three specimens from the Motmot tomb and Altar Q felids (+27.1, +26.9, and +26.8‰). This group was statistically distinct from the rest of the felid population (two-tailed t test, *p* < 0.001). Similarly, two individuals have values just below one standard deviation (+23.6 and +22.7‰, *p* < 0.001). Since the claws from Ruler 12 most likely originate from pelt remnants, it is reasonable to interpret that secondary faunal products were traded into Copan, as they exhibit higher δ^18^O_carbonate_ values. Later codices describe such trade networks at play in Mesoamerica, which list important tribute items including jaguar pelts traded from the Maya region to the Aztecs [108].

The three individuals that are above the range from Motmot and Altar Q assemblages must be interpreted more carefully, given that their extremely high C_4_ intake is indicative of captive behavior. The reservoir effect may explain the elevated δ^18^O values, caused by giving captive felids stored water in such reservoirs, or they may have been transported from distant regions. More conclusive evidence about where these felids originated would require additional analyses, such as strontium isotopes, to greatly enrich the sources of data available to investigators. Needless to say, the acquisition of these highly territorial and dangerous predators in the large quantities demanded by the commemoration event could have entailed a hazardous and difficult expedition to import them from afar.

## Conclusions

The isotope results demonstrate that an integrative approach to investigating and understanding the rich zooarchaeological assemblage at Copan significantly improved interpretive depth and nuance. Stable isotope analyses, in particular, exposed subtle changes in animal diet and mobility that zooarchaeological indicators alone did not detect. Unlike the Teotihuacan case study, there were no pathological indicators of captivity and the age distribution of the assemblage lacked the proportion of young individuals typical of a captive population. Yet the δ^13^C_collagen_, δ^13^C_carbonate_, and simple carbon models identified two distinct population clusters: 1) those fed on heavily C_4_ based diets, corresponding with the felids interred in Altar Q and Motmot deposits, and 2) felids and felid products—including some interred secondarily into the offering caches—with a predominance of C_3_ signatures indicative of a wild dietary pattern. Their δ^18^O values further substantiate the postulation that animals and their by-products were an important part of the expansive trade network established across the greater Copan Valley region and beyond, routing their carcasses from sites of capture to be processed into secondary products like pelts and venison and traded, or delivering them alive and intact to centers of state power for ritual usage and display.

The implication of this find is clear: Mesoamerican cultures manipulated (kept in captivity and transported across the landscape) a wide variety of animals ranging from those domesticated for food to highly specialized carnivores used for ritual purposes, and this practice was much more extensive than previously demonstrated. Until recently, academic scholarship concerning animal management practices in Mesoamerica had remained relatively undeveloped. This may be a consequence, in part, of the unusual diagnostic challenges arising from the spectacular and disruptive transformation of Mesoamerican ecology and terrain instigated by the European biotic invasion (e.g. livestock) [9,10], especially when contrasted with the relatively insignificant impact that the few Mesoamerican domesticates had on their Spanish adopters. Yet the increasing attention directed toward reconstructing pre-contact Mesoamerican practices for managing deer, rabbits, and other local animal resources has already proven fruitful, particularly when zooarchaeological and isotopic datasets are analyzed in concert [12,18,20,24]. The contribution of Copan data to this growing body of evidence enhances the potential for developing a more comprehensive Classic Mesoamerican resource model; one in which animals and animal products–as essential components of public and domestic rituals–are regularly procured, managed, and circulated.

Based on multiple congruent streams of evidence from the Motmot and Altar Q caches, we propose that felid procurement and captivity were practiced throughout the history of the Copan dynasty (A.D. 426–820). Other fauna, such as deer from both ritual and midden contexts, revealed patterns of variation in their zooarcheological and isotopic markers indicative of animals being traded across the Mesoamerican landscape. The deer-crocodilian collar and related evidence furnished by the shaman burial vividly depict elite usage of animal craft products to construct direct links to creatures from mythological realms usually restricted to royal personages [43]. Some of the midden elements found in other residential complexes bear characteristics which suggest they originated somewhere other than the local Copan Valley and should be tested with strontium isotope studies. The Motmot cache also featured animal products associated with other functions, such as food for the afterlife, and secondary products such as the bird elements distributed throughout the cache, which may have been incorporated in the assembly for their brilliant plumage.

This Classic period Mesoamerican site readily invites comparison with Teotihuacan. As with its mighty northern counterpart, excavation and analysis at Copan continues to yield a profusion of evidence telling the tale of a society deeply and intimately acquainted with the uses, benefits, and behaviors of the animals inhabiting their settlement as well as from beyond their local domain. Animals fulfilled a myriad of roles and functions at all social strata, and were especially significant components of Copan ritual practice and identity construction.

Further research into human-animal interactions at Copan, particularly when conducted using an integrated zooarchaeological and isotopic analysis methodology, provides an extraordinarily rich seam to mine for both broadly applicable field datasets as well as for the more focused archaeological insights uniquely expressed by this universal and fundamental feature common to all Mesoamerican lifeways.

## Supporting information

S1 Table Raw data of isotope analysis before data corrections. Yellow cells indicate samples that were dropped based on the diagenesis tests, d = dentine, e = enamel.
(XLSX)

## References

[1] Blanco A, Pérez G, Rodríguez B, Sugiyama N, Torres F, Valadez R. El Zoológico de Moctezuma ¿Mito o realidad? AMMVEPE. 2009;20: 29–39.

[2] Sugiyama N, Somerville AD, Schoeninger MJ. Stable Isotopes and Zooarchaeology at Teotihuacan, Mexico Reveal Earliest Evidence of Wild Carnivore Management in Mesoamerica. PLoS ONE. 2015;10: e0135635 10.1371/journal.pone.0135635 26332042 PMC4557940

[3] Ortner SB. On Key Symbols. Am Anthropol. 1973;75: 1338–46.

[4] Sugiyama N, Pérez G, Rodríguez B, Torres F, Valadez R. Animals and the State: The Role of Animals in State-Level Rituals in Mesoamerica In: McCarty SA, Arbuckle B, editors. Animals and Inequality in the Ancient World. Boulder: University Press of Colorado; 2014 pp. 11–31.

[5] Valadez RA. Perro Mexicano. México, D.F.: Universidad Nacional Autónoma de México; 1995.

[6] McKusick CR. Southwest Indian Turkeys Prehistory and Comparative Osteology. Globe, Arizona: Southwest Bird Laboratory; 1986.

[7] Thornton EK, Emery KF, Steadman DW, Speller C, Matheny R, Yang D. Earliest Mexican Turkeys (*Meleagris gallopavo*) in the Maya Region: Implications for Pre-Hispanic Animal Trade and the Timing of Turkey Domestication. PLoS ONE. 2012;7: e42630 10.1371/journal.pone.0042630 22905156 PMC3414452

[8] Thornton E, Emery KF, Speller C. Ancient Maya Turkey Husbandry: Testing Theories Through Stable Isotope Analysis. J Archaeol Sci Rep. 2016;10: 584–595.

[9] Crosby AW. The Columbian Exchange: Biological and Cultural Consequences of 1492. Westport, Conn.: Greenwood Pub. Co.; 1972.

[10] McClung de Tapia E, Sugiyama N. Conservando la Diversidad Biocultural de México: El Uso de Algunas Plantas y Animales en el Pasado y Presente. Arqueol Mex. 2012;XIX: 20–25.

[11] McKusick CR. Southwest Birds of Sacrifice. Globe, Arizona: Arizona Archaeological Society; 2001.

[12] Somerville AD, Nelson BA, Knudson KJ. Isotopic Investigation of Pre-Hispanic Macaw Breeding in Northwest Mexico. J Anthropol Archaeol. 2010;29: 125–135.

[13] Di Peso CC, Rinaldo JB, Fenner GJ. Casas Grandes: A Fallen Trading Center of the Gran Chichimeca 1st ed Dragoon and Flagstaff, Arizona: Amerind Foundation; Northland Press; 1974.

[14] López Luján L, Chávez Balderas X, Zúñiga-Arellano, Aguirre Molina A, Valentín Maldonado N. Entering the Underworld: Animal Offerings at the Foot of the Great Temple of Tenochtitlan In: McCarty SA, Arbuckle B, editors. Animals and Inequality in the Ancient World. Boulder: University Press of Colorado; 2014 pp. 33–61.

[15] Nicholson HB. Moctezuma’s Zoo. Pac Discov. 1955;4: 3–11.

[16] Corona Martínez E. Birds of the Pre-Hispanic Domestic Spheres of Central Mexico In: Götz CM, Emery KF, editors. The Archaeology of Mesoamerican Animals. Atlanta, Georgia: Lockwood Press; 2013 pp. 81–94.

[17] Valadez Azúa R. La Domesticación Animal. Mexico, D.F.: Universidad Naciona Autónoma de México, Instituto de Investigaciones Antropológicas; 2003.

[18] Somerville AD, Sugiyama N, Manzanilla LR, Schoeninger MJ. Animal Management at the Ancient Metropolis of Teotihuacan, Mexico: Stable Isotope Analysis of Leporid (Cottontail and Jackrabbit) Bone Mineral. PLoS ONE. 2016;11: e0159982 10.1371/journal.pone.0159982 27532515 PMC4988673

[19] Somerville AD, Sugiyama N, Manzanilla LR, Schoeninger MJ. Leporid Management and Specialized Food Production at Teotihuacan: Stable Isotope Data from Cottontail and Jackrabbit Bone Collagen. Archaeol Anthropol Sci. 2017;9: 83–97. 10.1007/s12520-016-0420-2

[20] White CD, Pohl MED, Schwarcz HP, Longstaffe FJ. Isotopic Evidence for Maya Patterns of Deer and Dog Use at Preclassic Colha. J Archaeol Sci. 2001;28: 89–107.

[21] Hamblin NL. Animal Use by the Cozumel Maya. Tucson, Arizona: University of Arizona Press; 1984.

[22] Hamblin NL, Rea AM. Isla Cozumel Archaeological Avifauna In: Pohl M, editor. Prehistoric Lowland Maya Environment and Subsistence Economy. Cambridge, Massachusetts: Peabody Museum of Archaeology and Ethnology Harvard University; 1985 pp. 175–192.

[23] Sharpe AE, Emery KF, Inomata T, Triadan D, Kamenov GD, Krigbaum J. Earliest Isotopic Evidence in the Maya Region for Animal Management and Long-distance Trade at the Site of Ceibal, Guatemala. Proc Natl Acad Sci. 2018; 201713880 10.1073/pnas.1713880115 29555750 PMC5889628

[24] Thornton EK. Reconstructing Ancient Maya Animal Trade Through Strontium Isotope (^87^Sr/^86^Sr) analysis. J Archaeol Sci. 2011;38: 3254–3263. 10.1016/j.jas.2011.06.035

[25] Aguilera C. Flora y Fauna Mexicana: Mitología y Tradiciones. México, D.F.: Editorial Everest Mexicana, S.A.; 1985.

[26] Careta MAN. Fauna Mexica: Naturaleza y Simbolismo. Netherlands: Research School CNWS, Leiden University; 2001.

[27] Stone AJ, Zender M. Reading Maya art: A Hieroglyphic Guide to Ancient Maya Painting and Sculpture. New York: Thames & Hudson; 2011.

[28] Benson EP. The Cult of the Feline: A Conference in Pre-Columbian Iconography. Washington, D.C.: Dumbarton Oaks Research Library and Collections; 1972.

[29] Saunders NJ. The Jaguars of Culture, Symbolizing Humanity in Pre-Columbian and American Societies. Doctoral Dissertation, University of Southampton. 1991.

[30] Saunders NJ. Icons of Power: Feline Symbolism in the Americas. London and New York: Routledge; 1998.

[31] Sugiyama N. La Noche y el Día en Teotihuacan. Artes México. 2016;121: 30–35.

[32] Stephens JL. Incidents of Travel in Central America, Chiapas, and Yucatan. New York: Harper & Brothers; 1841.

[33] Fash WL, Long KZ. Mapa Arqueológico del Valle de Copán. Introducción a la Arqueología de Copán, Honduras. Tegucigalpa, D.C.: Proyecto Arqueológico Copán, Secretaria de Estado en el Despacho de Cultura y Turismo; 1983.

[34] Bell EE, Canuto MA, Sharen RJ. Understanding Early Classic Copan. Philadelphia, PA: University of Pennsylvania Museum of Archaeology and Anthropology; 2004.

[35] Fash WL. Scribes, Warriors and Kings: The City of Copán and the Ancient Maya. London: Thames & Hudson Ltd; 1991.

[36] Fash WL. Towards a Social History of the Copán Valley In: Andrews EW, Fash WL, editors. Copán: The History of an Ancient Maya Kingdom. 1st ed Santa Fe: Oxford: School of American Research Press; James Currey; 2005 pp. 73–101.

[37] Willey GJ, Leventhal RM, Fash WL. Maya Settlement in the Copan Valley. Archaeology. 1978;31: 32–43.

[38] Fash WL, Williamson RV, Larios CR, Palka J. The Hieroglyphic Stairway and its Ancestors: Investigations of Copan Structure 10L-26. Anc Mesoam. 1992;3: 105–115.

[39] Fash WL, Fash B, Davis-Salazar K. Setting the Stage: Origins of the Hieroglyphic Stairway Plaza on the Great Period Ending In: Bell EE, Canuto MA, Sharer RJ, editors. Understanding Early Classic Copan. Philadelphia, PA: University of Pennsylvania Museum of Archaeology and Anthropology; 2004 pp. 65–83.

[40] Fash WL, Tokovinine A, Fash BW. The House of New Fire at Teotihuacan and its Legacy in Mesoamerica In: Fash WL, López Luján L, editors. The Art of Urbanism: How Mesoamerican Kingdoms Represented Themselves in Architecture and Imagery. Washington, D.C.: Dumbarton Oaks Research Library and Collection; 2009 pp. 201–229.

[41] Fash WL, Fash BW. Teotihuacan and the Maya: A Classic Heritage In: Carrasco D, Jones L, Sessions S, editors. Mesoamerica’s Classic Heritage: From Teotihuacan to the Aztecs. Boulder, Colorado: University Press of Colorado; 2000 pp. 433–464.

[42] Sharer RJ. Early Classic Royal Power in Copan: The Origins and Development of the Acropolis (ca. AD 250–600) In: Andrews EW, Fash WL, editors. Copán: the History of an Ancient Maya Kingdom. 1st ed Santa Fe: Oxford: School of American Research Press; James Currey; 2005.

[43] Sugiyama N, Fash WL, France CAM. Creating the cosmos, reifying power: A zooarchaeological investigation of corporal animal forms in the Copan Valley. Submitted to Camb Archaeol J. In Review;

[44] Buikstra JE, Price DT, Wright LE, Burton JH. Tombs from the Copan Acropolis: A Life History Approach In: Bell EE, Canuto MA, Sharer RJ, editors. Understanding Early Classic Copan. 1st ed Philadelphia: University of Pennsylvania Museum; 2004 pp. 185–205.

[45] Houston SD, Stuart D. The Way Glyph: Evidence for “Co-essences” Among the Classic Maya. Cent Maya Res. 1989; 1–16.

[46] Platt SG, Thorbjarnarson JB, Rainwater TR. Distribution of Morelet’s Crocodile (*Crocodylus moreletii*) in Southern Belize. Southwest Nat. 1999;44: 395–398. 10.2307/30055240

[47] Thorbjarnarson JB. American crocodile *Crocodylus acutus* In: Manolis SC, Stevenson C, editors. Crocodiles Status Survey and Conservation Action Plan. 3rd ed 2010 pp. 46–53.

[48] Stone AJ, Zender M. Reading Maya Art: A Hieroglyphic Guide to Ancient Maya Painting and Sculpture New York: Thames & Hudson; 2011.

[49] Stuart D. A Cosmological Throne at Palenque. www.mesoweb.com/stuart/notes/Throne.pdf; 2003.

[50] Saunders NJ. El Icono Felino en México: Fauces, Garras y Uñas. Arqueol Mex. 2005;12: 20–27.

[51] Ballinger DA, Stomper J. The Jaguars of Altar Q, Copán, Honduras: Faunal Analysis, Archaeology, and Ecology. J Ethnobiol. 2000;20: 223–236.

[52] Webster D, Freter A. Settlement History and the Classic Collapse at Copan: A Redefined Chronological Perspective. Lat Am Antiq. 1990;1: 66–85. 10.2307/971710

[53] Abrams EM, Rue DJ. The Causes and Consequences of Deforestation Among the Prehistoric Maya. Hum Ecol. 1988;16: 377–395.

[54] McNeil CL, Burney DA, Pigott Burney L. Evidence Disputing Deforestation as the Cause for the Collapse of the Ancient Maya Polity of Copan, Honduras. PNAS. 2009;107: 1017–1022. 10.1073/pnas.0904760107 20018691 PMC2824285

[55] Martin S, Grube N. Chronicle of the Maya Kings and Queens: Deciphering the Dynasties of the Ancient Maya. London: Thames & Hudson; 2000.

[56] Schaller GB, Drawshaw PGJ. Movement Patterns of Jaguar. Biotropica. 1980;12: 161–168.

[57] Rabinowitz AR, Nottingham BGJ. Ecology and Behaviour of the Jaguar (*Panthera onca*) in Belize, Central America. J Zool Lond A. 1986;210: 149–159.

[58] Behrensmeyer AK. Taphonomic and Ecologic Information from Bone Weathering. Paleobiology. 1978;4: 150–162.

[59] Collins LM. The Zooarchaeology of the Copan Valley: Social Status and the Search for a Maya Slave Class. Ph.D Dissertation, Harvard University. 2002.

[60] O’Leary MH. Carbon Isotopes in Photosynthesis: Fractionation Techniques May Reveal New Aspects of Carbon Dynamics in Plants. Bioscience. 1988;38: 328–336.

[61] Smith BN, Epstein S. Two Categories of ^13^C/^12^C Ratios for Higher Plants. Plant Physiol. 1971;47: 380–384. 16657626 10.1104/pp.47.3.380 PMC365873

[62] Ambrose SH, Norr L. Experimental Evidence for the Relationship of the Carbon Isotope Ratios of Whole Diet and Dietary Protein to Those of Bone Collagen and Carbonate In: Lambert JB, Grupe G, editors. Prehistoric Human Bone: Archaeology at the Molecular Level. Berlin; New York: Springer-Verlag; 1993 pp. 1–37.

[63] Froehle AW, Kellner CM, Schoeninger MJ. FOCUS: Effect of Diet and Protein Source on Carbon Stable Isotope Ratios in Collagen: Follow up to Warinner and Tuross (2009). J Archaeol Sci. 2010;37: 2662–2670. 10.1016/j.jas.2010.06.003

[64] Kellner CM, Schoeninger MJ. A Simple Carbon Isotope Model for Reconstructing Prehistoric Human Diet. Am J Phys Anthropol. 2007;133: 1112–1127. 10.1002/ajpa.20618 17530667

[65] Somerville AD, Fauvelle M, Froehle AW. Applying New Approaches to Modeling Diet and Status: Isotopic Evidence for Commoner Resiliency and Elite Variability in the Classic Maya Lowlands. J Archaeol Sci. 2013;40: 1539–1553. 10.1016/j.jas.2012.10.029

[66] Tieszen LL, Fagre T. Effect of Diet Quality and Composition on the Isotopic Composition of Respiratory CO_2_, Bone Collagen, Bioapatite, and Soft Tissue In: Lambert JB, Grupe G, editors. Prehistoric Human Bone: Archaeology at the Molecular Level. Berlin; New York: Springer-Verlag; 1993 pp. 121–155.

[67] Yoder C. Diet in Medieval Denmark: A Regional and Temporal Comparison. J Archaeol Sci. 2010;37: 2224–2236. 10.1016/j.jas.2010.03.020

[68] Ambrose SH. Effects of Diet, Climate and Physiology on Nitrogen Isotope Abundances in Terrestrial Foodwebs. J Archaeol Sci. 1991;18: 293–317.

[69] Hartman G. Are Elevated δ^15^N Values in Herbivores in Hot and Arid Environments Caused by Diet or Animal Physiology? Funct Ecol. 2011;25: 122–131.

[70] Warinner C, Garcia NR, Tuross N. Maize, Beans and the Floral Isotopic Diversity of Highland Oaxaca, Mexico. J Archaeol Sci. 2013;40: 868–873. 10.1016/j.jas.2012.07.003

[71] Hedges REM, Reynard LM. Nitrogen Isotopes and the Trophic Level of Humans in Archaeology. J Archaeol Sci. 2007;34: 1240–1251. 10.1016/j.jas.2006.10.015

[72] Minagawa M, Wada E. Stepwise Enrichment of ^15^N Along Food Chains: Further Evidence and the Relation Between δ^15^N and Animal age. Geochim Cosmochim Acta. 1984;48: 1135–1140.

[73] Schoeninger MJ, DeNiro MJ, Tauber H. Stable Nitrogen Isotope Ratios of Bone Collagen Reflect Marine and Terrestrial Components of Prehistoric Human Diet. Science. 1983;220: 1381–1383. 6344217 10.1126/science.6344217

[74] Schoeninger MJ, DeNiro MJ. Nitrogen and Carbon Isotopic Composition of Bone Collagen from Marine and Terrestrial Animals. Geochim Cosmochim Acta. 1984;48: 625–639. 10.1016/0016-7037(84)90091-7

[75] Rawlings TA, Driver JC. Paleodiet of Domestic Turkey, Shields Pueblo (5MT3807), Colorado: Isotopic Analysis and its Implications for Care of a Household Domesticate. J Archaeol Sci. 2010;37: 2433–2441.

[76] Hobson KA. Tracing Origins and Migration of Wildlife Using Stable Isotopes: A Review. Oecologia. 1999;120: 314–326. 10.1007/s004420050865 28308009

[77] Knudson KJ, Price TD. Utility of Multiple Chemical Techniques in Archaeological Residential Mobility Studies: Case Studies from Tiwanaku and Chiribaya affiliated Sites in the Andes. Am J Phys Anthropol. 2007;132: 25–39. 10.1002/ajpa.20480 17063464

[78] Luz B, Kolodny Y. Oxygen Isotope Variation in Bone Phosphate. Appl Geochem. 1989;4: 317–323. 10.1016/0883-2927(89)90035-8

[79] Bowen GJ. Isoscapes: Spatial Pattern in Isotopic Biogeochemistry. Annu Rev Earth Planet Sci. 2010;38.

[80] Price D, Manzanilla LR, Middleton WD. Immigration and the Ancient City of Teotihuacan in Mexico: A Study Using Strontium Isotope Ratios in Human Bone and Teeth. J Archaeol Sci. 2000;27: 903–913.

[81] Price TD, Burton JH, Wright LE, White CD, Longstaffe F. Victims of Sacrifice: Isotopic Evidence for Place of Origin In: Tiesler V, Cucina A, editors. New Perspectives on Human Sacrifice and Ritual Body Treatment in Ancient Maya Society. New York: Springer; 2007 pp. 263–292.

[82] White CD, Price D, Longstaffe FJ. Residential Histories of the Human Sacrifices at the Moon Pyramid, Teotihuacan: Evidence from Oxygen and Strontium Isotopes. Anc Mesoam. 2007;18: 159–172.

[83] White CD, Spence MW, Longstaffe FJ, Law KR. Testing the Nature of Teotihuacan Imperialism at Kaminaljuyu Using Phosphate Oxygen-Isotope Ratios. J Anthropol Res. 2000;56: 535–558.

[84] Wassenaar LI, Van Wilgenburg SL, Larson K, Hobson KA. A Groundwater Isoscape (δD, δ^18^O) for Mexico. J Geochem Explor. 2009;102: 123–136.

[85] Price TD, Burton JH, Sharer RJ, Buikstra JE, Wright LE, Traxler LP, et al Kings and Commoners at Copan: Isotopic Evidence for Origins and Movement in the Classic Maya Period. J Anthropol Archaeol. 2010;29: 15–32. 10.1016/j.jaa.2009.10.001

[86] Price TD, Nakamura S, Suzuki S, Burton JH, Tiesler V. New Data on Maya Mobility and Enclaves at Classic Copan, Honduras. J Anthropol Archaeol. 2014;36: 32–47.

[87] Scherer AK, de Carteret A, Newman S. Local Water Resource Variability and Oxygen Isotopic Reconstructions of Mobility: A Case Study from the Maya Area. J Archaeol Sci Rep. 2015;2: 666–676. 10.1016/j.jasrep.2014.11.006

[88] Cerling TE, Sharp ZD. Stable Carbon and Oxygen Isotope Analysis of Fossil Tooth Enamel Using Laser Ablation. Palaeogeogr Palaeoclimatol Palaeoecol. 1996;126: 173–186. 10.1016/S0031-0182(96)00078-8

[89] Wright LE, Schwarcz HP. Stable Carbon and Oxygen Isotopes in Human Tooth Enamel: Identifying Breastfeeding and Weaning in Prehistory. Am J Phys Anthropol. 1998;106: 1–18. 10.1002/(SICI)1096-8644(199805)106:1<1::AID-AJPA1>3.0.CO;2-W 9590521

[90] Longin R. New Method of Collagen Extraction for Radiocarbon Dating. Nature. 1971;230: 241–242. 10.1038/230241a0 4926713

[91] France CAM, Owsley DW, Hayek L-AC. Stable Isotope Indicators of Provenance and Demographics in 18th and 19th Century North Americans. J Archaeol Sci. 2014;42: 356–366. 10.1016/j.jas.2013.10.037

[92] Schimmelmann A, Albertino A, Sauer PE, Qi H, Molinie R, Mesnard F. Nicotine, acetanilide and urea multi-level ^2^H-, ^13^C- and ^15^N-abundance reference materials for continuous-flow isotope ratio mass spectrometry. Rapid Commun Mass Spectrom. 2009;23: 3513–3521. 10.1002/rcm.4277 19844968

[93] Bryant JD, Koch PL, Froelich PN, Showers WJ, Genna BJ. Oxygen Isotope Partitioning Between Phosphate and Carbonate in Mammalian Apatite. Geochim Cosmochim Acta. 1996;60: 5145–5148.

[94] Garvie-Lok SJ, Varney TL, Katzenberg MA. Preparation of Bone Carbonate for Stable Isotope Analysis: The Effects of Treatment Time and Acid Concentration. J Archaeol Sci. 2004;31: 763–776. 10.1016/j.jas.2003.10.014

[95] Pellegrini M, Snoeck C. Comparing Bioapatite Carbonate Pre-treatments for Isotopic Measurements: Part 2—Impact on Carbon and Oxygen Isotope Compositions. Chem Geol. 2016;420: 88–96. 10.1016/j.chemgeo.2015.10.038

[96] Ambrose SH. Preparation and Characterization of Bone and Tooth Collagen for Isotopic Analysis. J Archaeol Sci. 1990;17: 431–451.

[97] DeNiro MJ. Postmortem Preservation and Alteration of In Vivo Bone Collagen Isotope Ratios in Relation to Palaeodietary Reconstruction. Nature. 1985;317: 806–809. 10.1038/317806a0

[98] Wright LE, Schwarcz HP. Infrared and Isotopic Evidence for Diagenesis of Bone Apatite at Dos Pilas, Guatemala: Palaeodietary Implications. J Archaeol Sci. 1996;23: 933–944. 10.1006/jasc.1996.0087

[99] Smith CI, Nielsen-Marsh CM, Jans MME, Collins MJ. Bone Diagenesis in the European Holocene I: Patterns and Mechanisms. J Archaeol Sci. 2007;34: 1485–1493. 10.1016/j.jas.2006.11.006

[100] Nielsen-Marsh CM, Smith CI, Jans MME, Nord A, Kars H, Collins MJ. Bone Diagenesis in the European Holocene II: Taphonomic and Environmental Considerations. J Archaeol Sci. 9;34: 1523–1531. 10.1016/j.jas.2006.11.012

[101] Weiner S, Bar-Yosef O. States of Preservation of Bones from Prehistoric Sites in the Near East: A Survey. J Archaeol Sci. 1990;17: 187–196. 10.1016/0305-4403(90)90058-D

[102] Warinner C, Tuross N. Alkaline Cooking and Stable Isotope Tissue-Diet Spacing in Swine: Archaeological Implications. J Archaeol Sci. 2009;36: 1690–1697.

[103] Webb EC, White CD, Longstaffe FJ. Investigating Inherent Differences in Isotopic Composition Between Human Bone and Enamel Bioapatite: Implications for Reconstructing Residential Histories. J Archaeolgocial Sci. 2014;50: 97–107.

[104] Schoeninger MJ. Trophic Level Effects on ^15^N/^14^N and ^13^C/^12^C Ratios in Bone Collagen and Strontium Levels in Bone Mineral. J Hum Evol. 1985;14: 515–525. 10.1016/S0047-2484(85)80030-0

[105] Schwarcz HP. Some Theoretical Aspects of Isotope Paleodiet Studies. J Archaeol Sci. 1991;18: 261–275. 10.1016/0305-4403(91)90065-W

[106] Linares OF. “Garden Hunting” in the American Tropics. Hum Ecol. 1976;4: 331–349. 10.2307/4602380

[107] Grigione MM, Beier P, Hopkins RA, Neal D, Padley WD, Schonewald CM, et al Ecological and Allometric Determinants of Home-range Size for Mountain Lions (*Puma concolor*). Anim Conserv. 2002;5: 317–324.

[108] Berdan F, Rieff Anawalt P, editors. The Codex Mendoza. Berkeley: University of California Press; 1992.

